# New PCL/PEC Blends: In Vitro Cell Response of Preosteoblasts and Human Mesenchymal Stem Cells

**DOI:** 10.3390/biology11081201

**Published:** 2022-08-10

**Authors:** Jesus L. Pablos, Mónica Cicuéndez, María Hernández-Rivas, Fernando Catalina, María Vallet-Regí, Teresa Corrales

**Affiliations:** 1 Departamento de Química Macromolecular Aplicada, Grupo de Fotoquímica, Instituto de Ciencia y Tecnología de Polímeros, C.S.I.C., Juan de la Cierva 3, 28006 Madrid, Spain; 2 Departamento de Química en Ciencias Farmacéuticas, Facultad de Farmacia, Universidad Complutense de Madrid, UCM, 28040 Madrid, Spain; 3 Networking Research Center on Bioengineering, Biomaterials and Nanomedicine, CIBER-BBN, 28040 Madrid, Spain

**Keywords:** PCL/PEC blends, biocompatible polymers, cell proliferation, differentiation, tissue engineering, bone regeneration

## Abstract

**Simple Summary:**

A topic of great relevance is the design of new biocompatible materials for their application in tissue engineering. The preparation of new polycaprolactone (PCL)/poly (ethylene carbonate) (PEC) blends has resulted in a series of new biomaterials obtained in a simple manner, which favor cell proliferation and subsequent differentiation using two different cell lines, the osteoblastic cell line (MC3T3-E1) and human mesenchymal cells (hMSCs), whose results indicate that these materials could be used in future to carry out 3D forming with the purpose of testing their potential application in tissue engineering for bone regeneration through “in vivo” assays.

**Abstract:**

In this study, new blends of PCL/PEC have been prepared in an easy manner by casting with the objective of obtaining new biomaterials to apply to tissue engineering and bone regeneration. The PCL/PEC blends obtained, together with neat polymer blends, were characterized by infrared spectroscopy (FTIR), atomic force microscopy (AFM), scanning electron microscopy (SEM), differential scanning calorimetry (DSC) and thermogravimetric analysis (TGA). This full characterization is the key to disentangle the miscibility, which means good compatibility, of the polymer blends used in this work. The addition of increasing amounts of PEC, has shown in the new biomaterials obtained, a remarkable improvement in relation with the mechanical properties (manageable materials) and above all, in terms of an increase in their hydrophilic character with respect to the PCL neat polymer. The improvement of all these properties is reflected in their biological properties. With these thoughts in mind, the blends obtained were tested through the assessment of several biological parameters such as cell viability, proliferation, and differentiation of both the MC3T3-E1 osteoblastic cell line and hMSCs to evaluate their cell response to different polymer membranes aimed at bone tissue regeneration. “In vitro” biocompatibility methods have been chosen rather than in vivo studies due to their lower cost, faster procedure time, and minimum ethical concerns, and because it was the first time that the biological effects of these blends were studied. The results show that the PCL/PEC blends obtained, with tunable properties in terms of hydrophilic character and hydrolytic degradation, may be regarded as good candidates to perform “in vivo” tests and check their real-life applicability for bone regeneration. The polymer acronym (the weight percentage in the sub index) is PCLx/PECy as noted in table one with the summary of compositions.

## 1. Introduction

One of the best designers in tissue engineering is nature. In this regard, there are multiple examples of natural designs that range from osseous structures, with high strength and sufficient flexibility without becoming brittle, to veins and arteries, which are elastomeric and flexible without being feeble. However, despite the healing capacity of the human body, the appearance of lesions and pathologies cannot be excluded. In this context, the field of research of tissue engineering must be highlighted, whose general purpose is to improve the quality of life in human beings, together with other more relevant aspects such as the reduction in costs when treating diseases, and the improvement, in general, of health care. An important approach in this area is the use of polymers as biomaterials to regenerate functional tissues or medical devices for implants. From the first syringe made with synthetic materials in 1955 [1] to the more and more sophisticated complex polymeric materials, this interdisciplinary field has experienced exponential growth since the 1990s, but much more still needs to be accomplished through the development of new materials with potential applications in regenerative tissue engineering in general, and, in particular, for bone regeneration. It must be noted that, since the early 1960s, synthetic polymers have been an important part of contemporary society. In addition to the well-known applications in multiple fields of daily life of polymers, it is necessary to point out that one of the greatest revolutions has transpired in the field of medicine [2].

Polymers used as biomaterials must possess a combination of specific features, such us adequate mechanical properties and convenient topography, all directed towards the improvement of cell adhesion. Furthermore, biomaterials must have the ability to stimulate the cell diversity and differentiation for renewing damaged tissues, avoiding harmful effects in living tissues along with good long-term biocompatibility in vivo [3,4,5]. In summary, the main purpose of this field is to develop functional substitutes for injured tissues with a specific criterion in terms of biocompatibility, biodegradability, maintaining suitable mechanical properties and, overall, allowing adhesion, cellular growth and proliferation followed by differentiation of cells on their surfaces, to achieve the possibility to be fused with the body’s own tissue in combination with nontoxicity [6].

One of the most popular synthetic biocompatible polymers is polycaprolactone (PCL) [7,8]. This polymer, defined as a semi-crystalline aliphatic biodegradable polyester [9,10], can be found in a long number of medical devices, and it is considered as one of the most investigated biocompatible synthetic aliphatic polyesters due to its excellent properties, which allows its application in the biomedical field, such as for drug delivery systems or tissue engineering [11]. Among its properties, its biocompatibility and ductility should be highlighted due to a low glass transition temperature (Tg) of −60 °C, which results in easy processability by extrusion, melt spinning or injection moulding. In contrast, it has relatively low mechanical strength, which can be a limiting factor in some practical applications. Nevertheless, it must be pointed out that applications of PCL [12] could be limited in terms of its degradation and resorption kinetics in a physiologic environment, showing a relatively low weight loss (<5%) in the first 25 weeks [13], which is considerably slower than other aliphatic polyesters on account of their hydrophobic character and high crystallinity index that limit the capacity of water to enter in the polymer network. This fact implies that its potential applications in a biomedical field overall are restricted [14,15,16].

Despite this, there are multiple studies where PCL is used as a biomaterial for bone repair itself or following the strategy of blending with another biocompatible synthetic polymer [17,18]. This is a useful method for obtaining a desirable combination of properties that are often missing in the single polymers individually or together with other advantages in terms of cost effectiveness and low preparation time by changing the blend composition. An example of this strategy is the obtention of PCL and PMMA (polymethyl methacrylate) blends when considering biomedical applications [19], that make use of the properties of PMMA, a rigid polymer with good biocompatibility, and one of the often-used polymers in biomedical engineering (contact lenses or bone implants) [20] with successful cell migration and proliferation [21]. This is frequently used for permanent tissue substitution surgery, despite not being biodegradable.

In recent years, another biocompatible polymer, PEC, has emerged as an alternative biodegradable polymer. PEC is an aliphatic polycarbonate that possesses good biodegradability and biocompatibility in the in vivo environment which has been extensively studied [22], and it is one of the few polymers reported which exhibits a rapid bio absorption in vivo through enzymatic activity without undesirable side effects [23,24]. This indicates that PEC and its degradation subproducts are biocompatible and induce wound healing responses [25]. An absence of any commercial medical application of PEC can be observed, which must be attributed to several factors such as their physical properties and difficulties in the polymer processing. For this reason, PEC is presented as an alternative biodegradable polymer, forming blends with other biocompatible polymers, such as polylactic acid (PLA) [26].

Considering all the data described above, this work has been approached as a proof of concept by preparing new polymeric blends based on PCL/PEC, by varying the proportion between them through the easy means of the casting method, since molecular structures of PCL and PEC are similar, which could enhance their miscibility and therefore compatibility. This work involves the preparation of new blends, so far unexplored to the best of our knowledge, and presents a basis of concept for future work with other functionalized polycarbonates. In this manner, it has been possible to study, firstly, the influence of PEC addition to the proposed blends by analyzing the changes in the physical and thermal properties, along with the study of the hydrolysis of the blends in alkaline solutions.

The study of the variation in these properties is of great importance since they are key aspects in their biological properties towards different cell lines. It must be stressed that each one has specific characteristics in terms of its expression of cell surface markers, long-term in vitro culturing, in vitro differentiation, potential, immunomodulatory features, and its homing capacity, among others. Therefore, this fact means that the same biomaterial can present different cellular responses depending on the cell type used. For this reason, it is important to carry out comparative studies using different cell types related to the final biomedical application, for which the candidate biomaterial will be intended. Therefore, in a second stage of this work, a comparative study of the cellular response to different compositions of blends obtained has been carried out using MC3T3-E1 osteoblastic cell line and hMSCs. Specifically, the viability, proliferation, and differentiation through the intracellular activity alkaline phosphatase (ALP) at different culture times have been studied to uncover their potential as new biomaterial and their possible implementation in tissue regeneration engineering for bone regeneration as an initial “in vitro” test.

## 2. Materials and Methods

### 2.1. Materials and Reagents

All materials and solvents were commercially available and used as received unless otherwise indicated. The following materials and solvents were used: QPAC^®^25 poly (ethylene carbonate) (PEC, Empower Materials, New Castle, DE, USA, Granulate form), sodium hydroxide pellets (PanReac, 100%), polycaprolactone pellets (PCL, Sigma-Aldrich, Darmstadt, Germany, Mn average 80,000), chloroform (Scharlau, reagent grade), MilliQ water.

In this study, two different cell lines were used. They were obtained from two sales companies: Mouse osteoblastic cell line (MC3T3-E1) were obtained from Sigma-Aldrich and were isolated from mouse bone (Mouse C57BL/6 calvaria, Phenotype: Adherent, Karyotype: Not specified, Morphology: Fibroblast-like). hMSCs were obtained from Lonza Sales Ltd. (Basel Stücki, Switzerland), material number PT-2501 and Batch N°: 19TL168853.

### 2.2. Membrane Preparation

Membranes with different percentages of PCL/PEC (ranging from 50 wt% to 10 wt% of PEC together with neat polymers, PCL and PEC, Table 1) were prepared by casting (solvent evaporation at controlled temperature).

For this purpose, 200 mg of polymer mixture, dissolved in 1.5 mL of chloroform, was used. This mixture was placed in Teflon molds and allowed to evaporate at rt to obtain the final materials. In each material evaluated, the indicated amount of each of the polymers was weighed to obtain a total weight of 200 mg (Table 1). The general procedure for the preparation of the materials was as follows:

First, the appropriate amount of PEC was dissolved in 1.5 mL of chloroform. To achieve a homogeneous solution of the polymer, heating and sonicating cycles were combined until the total dissolution of the polymer. Once the PEC was dissolved and the solution was cold, the required quantity of PCL was added to prepare each membrane. After sonicating for 10 min, a homogeneous and transparent solution of both polymers was obtained. This solution was poured over into a Teflon mold and the solvent was allowed to evaporate in these controlled conditions for 24 h at room temperature. In this manner, both the solid and manageable materials were obtained. Finally, the membranes were demolded and stored for further analysis. A brief narrative summary of all materials obtained, along with their compositions and preparation conditions, is showed in Table 1.

### 2.3. Membrane Characterization: Instrumentation and Methods

Attenuated total reflectance/FT-infrared spectroscopy (ATR-FTIR) was used to characterize PCL/PEC membranes of different compositions. ATR-FTIR spectra were registered using a Perkin Elmer BXFTIR Spectrometer coupled with a MIRacle™ ATR accessory, from PIKE Technologies.

Thermal properties of PCL/PEC blends were analyzed by differential scanning calorimetry (DSC) and thermogravimetric analysis (TGA). DSC was performed on a METTLER DSC-823e instrument which was previously calibrated with an indium standard. Membrane samples (4 mg) were placed in aluminum DSC pans, and the thermal history was erased by heating to 100 °C at 10 °C/min in a first scan and maintaining the temperature for 7 min. Then, the samples were cooled to −60 °C and the temperature was increased to 100 °C at a rate of 10 °C/min under a nitrogen atmosphere in a second scan to determine the melting temperature and crystallinity index of the PCL/PEC membranes. Crystallinities of the blends were calculated using the standard enthalpy of PCL (ΔH_m_ = 139.5 J/g) with the following equation: χ_c_ = (ΔH_m_/ΔH_m_^0^) × 100. TGA was carried out in a TA Q-500 TA Instrument under a nitrogen atmosphere, from 25 to 800 °C at a heating rate of 10 °C/min. The T_5_ (defined as the temperature of degradation with a weight loss of 5%) and T_max_ degradation temperature were determined.

Surface characterization was undertaken using contact angle and atomic force microscopy. The contact angle on the surfaces was measured by the “sessile drop” method using milliQ water as the wetting solvent at 25 °C and a CAM200 KSV tensiometer. Sessile drop contact angle measurements were performed dynamically; an initial liquid drop of a radius of about 0.3 cm was carefully deposited on the surface using a motor-driven syringe to pump liquid steadily into the sessile drop from below the surface, and to ensure that the drop will increase in the center of the image field. Tapping-mode atomic force microscopy (TM-AFM) measurements were conducted in air with a Nanoscope IV system (Digital Instruments) with a triangular micro-fabricated cantilever with a length of 115–135 μm, 1–10 Ohm cm phosphorous (n)-doped Si pyramidal tip, and a nominal spring constant of 20–80 Nm^−1^. A resonance frequency of the cantilever typically at 275 KHz was chosen for the tapping mode oscillation. Moderate tapping forces were used by setting the set-point ratio between 0.6 and 0.7. The AFM images were obtained with a maximum scan range of 20 × 20 μm^2^.

An alkaline hydrolysis test was performed using films of controlled thickness of approximately 500 μm in 20 mL 1M NaOH aqueous solution. The films were placed in a glass vial filled with this NaOH solution, and the vial was covered. Hydrolysis was performed at 37 °C for a predetermined period of 96 h. After hydrolysis, the films were washed with distilled water at room temperature and the NaOH solution was wiped off the surface, then the film was quickly weighed. The resulting films were dried at room temperature for 24 h, then they were weighed (W_dried_). The weight loss (W_loss_) was calculated using Equation (1).

W_Loss_ = (W_Dried_ − W_Initial_)/W_Initial_ × 100 (1)

Film surfaces before and after hydrolysis treatment were coated with approximately 3 nm of gold/palladium using a PolaronSC7640 sputter coater and examined by scanning electron microscopy (SEM), employing a SEM Philips XL30 model.

### 2.4. In Vitro Biocompatibility Assays

In vitro biocompatibility assays were carried out in terms of viability, cellular proliferation and differentiation of all blends obtained. First, membranes were immersed in the cultured medium so that the materials can be swelled and cleaned (37 °C and 5% CO_2_). After 16 h, the medium was removed, and membranes were washed with PBS. For cell viability and proliferation assays, the MC3T3-E1 osteoblastic cell line was seeded at a density of 10^5^ cells/mL. Then, they were cultured in 2 mL/well of alpha-Minimum Essential Medium (alpha-MEM, Sigma Chemical Company, St. Louis, MO, USA). Cells were maintained for 1 and 7 days at 37 °C under a 5% CO_2_ atmosphere. After 1 and 7 days, materials with cells on the surface were treated with 0.25% EDTA-trypsin solution for 10 min. Cell number and viability percentages were obtained from cell counter equipment using trypan blue.

hMSCs were seeded at a density of 10^5^ cells/mL. Then, they were cultured in 2 mL/well of Mesenchymal Stem Cell Growth Medium BulletKit (Lonza) and maintained for 1 day and 7 days at 37 °C under a 5% CO_2_ atmosphere. Proliferation was measured using the WST-8 Cell Proliferation Kit by obtaining the values of absorbance at 450 nm. Colorimetric measurement at 450 nm allows quantification of viable cells. The WST-8 Cell Proliferation Kit is a colorimetric assay for the determination of viable cell number and for studying induction or inhibition of cell proliferation in vitro. This assay kit is based on the cellular reduction in the tetrazolium salt WST-8 (2-(2-methoxy-4-nitrophenyl)-3-(4-nitrophenyl)-5-(2,4-disulfophenyl)-2H-tetrazolium, monosodium salt) into a highly water-soluble, orange-colored formazan dye upon reduction in the presence of an electron carrier. As opposed to the MTT assay, no solubilization process is required since this formazan does not require solvation: the WST-8 is soluble in the tissue culture medium.

For differentiation cell assays, MC3T3-E1 osteoblastic cell line and hMSCs were seeded at a density of 10^5^ cells/mL. Then, MC3T3-E1 pre-osteoblasts were cultured in 2 mL/well of differentiation culture medium: alpha-Minimum Essential Medium (alpha-MEM, Sigma Chemical Company, St. Louis, MO, USA) supplemented with 10% FBS, 50 g/mL β-glycerolphosphate, 10 mM L-ascorbic acid, 1 mM L-hglutamine, penicillin, and streptomycin and human mesenchymal stem cells were cultured in 2 mL/well of Human Mesenchymal Stem Cell Osteogenic Differentiation Medium BulletKit (Lonza). Then, cells were maintained for 7 days at 37 °C under a 5% CO_2_ atmosphere. The alkaline phosphatase (ALP) activity was used as the key differentiation marker in assessing expression of the osteoblast phenotype. After 7 days of culture, cells were lysed by 3 consecutive freezing/thawing cycles. Lysates were incubated with 10 mM *p*NPP solution in the culture conditions and then the reactions were stopped by adding 2 M NaOH. Cellular ALP activities were measured spectrophotometrically, by measuring the increase in absorbance at 405 nm accompanying the production of *p*-nitrophenol and normalized by the cell protein content, which was determined by measuring the absorbance at 540 nm.

For matrix mineralization assay, the detection of calcium deposits was performed in MC3T3-E1 preosteoblasts by alizarin red staining. After 10 days of culture, cells were washed with PBS and then fixed with glutaraldehyde (10% PBS) for 1 h. Cell cultures were stained with 40 mM alizarin red in distilled water (pH 4.2) for 45 min at room temperature. Subsequently, cell monolayers were washed gently with distilled water and the calcium deposits were dissolved with 10% cetylpyridinum chloride in 10 mM sodium phosphate, pH 7.0, and absorbance was measured at 620 nm.

In general, for all biological assays, polymer materials were placed in 6-well plates previously and finally, in a new 6-well plates after cell exposure, for ensuring that we only considered cells which have growth in the material surface.

## 3. Results

In this study, membranes based on blends of PCL and PEC, were prepared and named using the polymer acronym with the weight percentage in the sub index, PCL_x_/PEC_y_, Table 1. The membranes were characterized by FTIR spectroscopy and the effect of the polymer composition in the blend were evaluated in terms of thermal properties, morphology, hydrolytic degradation, and cell viability.

### 3.1. FTIR Characterization

**Figure 1:**
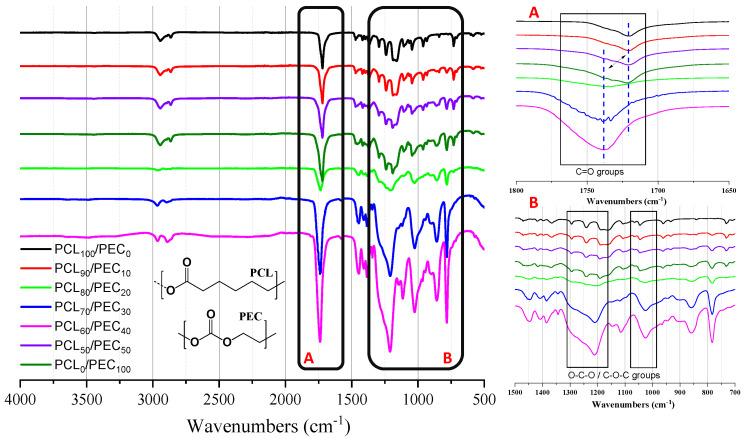
(**Left**) FTIR spectra of the PCL and PEC materials, along with the different blends obtained. (**Right**) detail amplified of the region of 1700–1800 cm^–1^ (**A**) of carbonyl groups, and the region of 1300–1100 cm^–1^ (**B**) of stretching bands of O-C-O and C-O-C groups.

All membranes were characterized by FTIR, Figure 1, with two priority targets: firstly, the confirmation of the presence of the two polymers, PEC and PCL, in the blends with different compositions, and secondly, with the purpose of determining the interactions between PEC and PCL phases, which is reflected in the good miscibility and compatibility of the blends.

Figure 1 shows the FTIR spectra of the PCL and PEC membranes, along with the different compositions obtained. In the region of 1700–1800 cm^−1^, the stretching vibrational mode of the -C=O group can be observed, where the carbonyl peaks of neat PEC and PCL materials appear at 1738 cm^−1^ and 1719 cm^−1^, respectively [27,28]. For the blends, the increasing addition of PCL (up to 90% weight) is reflected in a decrease in intensity of the PEC carbonyl peak at 1738 cm^−1^ and the progressive shift to a lower wavenumber. On the other hand, the PCL carbonyl peak at 1719 cm^−1^ was seen to decrease and shift to a higher wavenumber when PEC increased in the blend. In addition, in the region of 1300–1100 cm^−1^, the analysis of strectching bands of O-C-O and C-O-C groups in blends showed a shift with respect to the spectra of the neat polymers.

### 3.2. Thermal Properties

#### 3.2.1. Thermogravimetric Analysis (TGA)

**Figure 2:**
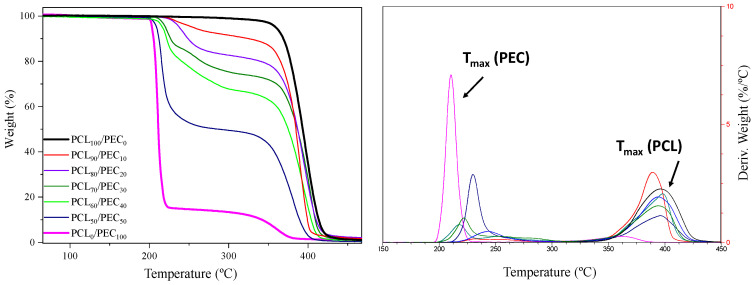
Thermogravimetric analysis (TGA), curves obtained for all materials obtained. (**Left**) graph shows weight loss (%) vs. temperature. (**Right**) graph shows the derivative weight loss curve (DTGA).

Thermal degradation of neat PCL and PEC together with the PCL/PEC blends obtained has been studied by determining the weight loss of each sample upon linearly increasing the temperature in the oxygen atmosphere by conventional thermogravimetric analysis (TGA); curves are shown in Figure 2. Furthermore, Table 2 sets out the thermogravimetric data for all membranes, in terms of the temperature at 5 wt% weight loss (T_5_) and the maximum decomposition temperature (T_max_) obtained from the derivative weight loss curve (DTGA). It can be observed in thermograms for neat PCL and PEC membranes that T_max_ occurred at 398 °C and 210 °C, respectively; as can be observed in Figure 2 and Table 1, the T_max_ in the blends take place between those of pure PCL and PEC. The addition of the increasing amount (wt%) of PCL in the blends improved the thermal stability of PEC and raised the values of T_5_ and T_max_ above those of neat PEC.

#### 3.2.2. Differential Scanning Calorimetry (DSC) Measurements

**Figure 3:**
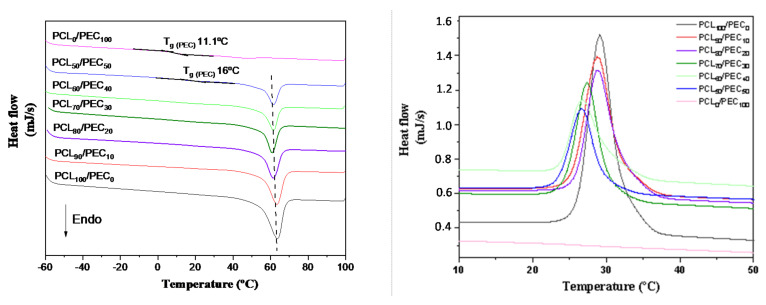
DSC curves obtained by heating to 100 °C at 10 °C/min in a first scan and maintaining the temperature for 7 min. Then, the samples were cooled to −60 °C (**right graph**) and the temperature was increased to 100 °C at 10 °C/min rate under nitrogen atmosphere in a second scan (**left graph**) to determine the melting temperature and crystallinity index of the PCL/PEC membranes.

The thermal properties of PEC, PCL and their blends were studied by DSC, Figure 3. PEC exhibited a Tg at 11 °C, and as a consequence of the addition of PCL in blends, the Tg value increased to 16 °C in PCL_50_/PEC_50_; this is not negligible for higher percentages of PCL. Additionally, it can also be noted that the melting temperature of PCL (Tm) and the crystallisation temperature (Tc) decreased slighltly to lower temperatures with the addition of PEC to the blends (Figure 3). Additionally, the calculated crystalllinity index decrased to lower values for the blends as the PEC is incorporated in the blends, from 50.7% for PCL to 19.5% for PCL_50_/PEC_50_, Table 3.

### 3.3. AFM Studies

**Figure 4:**
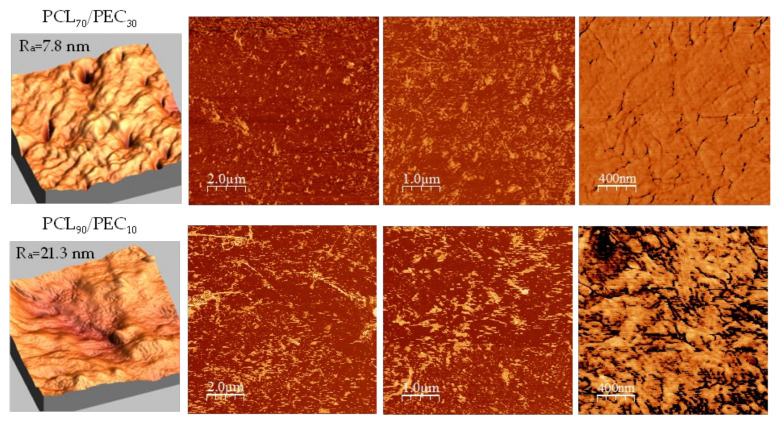
AFM morphology and phase images of PCL_70_/PEC_30_ and PCL_90_/PEC_10_ membranes.

Atomic force microscopy was performed to better assess the morphology and compatibility of the PCL/PEC blends. Figure 4 shows AFM images of PCL_90_/PEC_10_ and PCL_70_/PEC_30_ membranes.

In general, it has been observed that surfaces of blends prepared have a low roughness average (7.8 nm and 21.3 nm for PCL_70_/PEC_30_ and PCL_90_/PEC_10_, respectively). By analyzing the phase images, the miscibility of the polymers can be confirmed in terms of the absence of microstructures and phase separation with a continuous morphology. However, it must be pointed out that the incorporation of PEC in the blends has an influence on the growth of PCL crystals; in the case of PCL_70_/PEC_30_, the crystal size decreases, and smaller homogeneously dispersed crystalline entities are observed with respect to those in PCL_90_/PEC_10_, which would agree with the slightly lower roughness average in PCL_70_/PEC_30_.

### 3.4. Alkalyne Hydrolisis

Hydrolytic degradation of neat polymers PCL and PEC and the corresponding blends was undertaken. That process is considered to be a bulk erosion process, which is linked with a decrease in the molecular weight.

**Figure 5:**
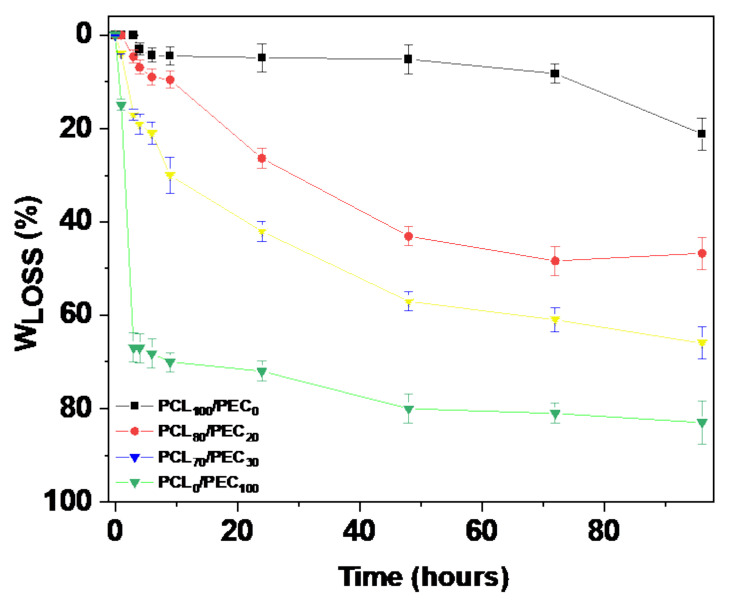
Weight loss (%) of of neat polymers PCL and PEC and the corresponding blends as a function of time immersed in 1M NaOH solution in the hydrolytic degradation study.

Figure 5 shows the weight loss (W_loss_) of the different membranes as a function of time immersed in the 1M NaOH solution in the hydrolysis test. The neat PEC polymer are almost completely hydrolysed in the alkaline solution after 96 h, whereas PCL hydrolysis is quite low in comparison with PEC. Both of the blends studied were clearly hydrolysed faster than the neat PCL, and the weight loss reaches values of 50% and 68% for PCL_80_/PEC_20_ and PCL_70_/PEC_30_, respectively.

**Figure 6:**
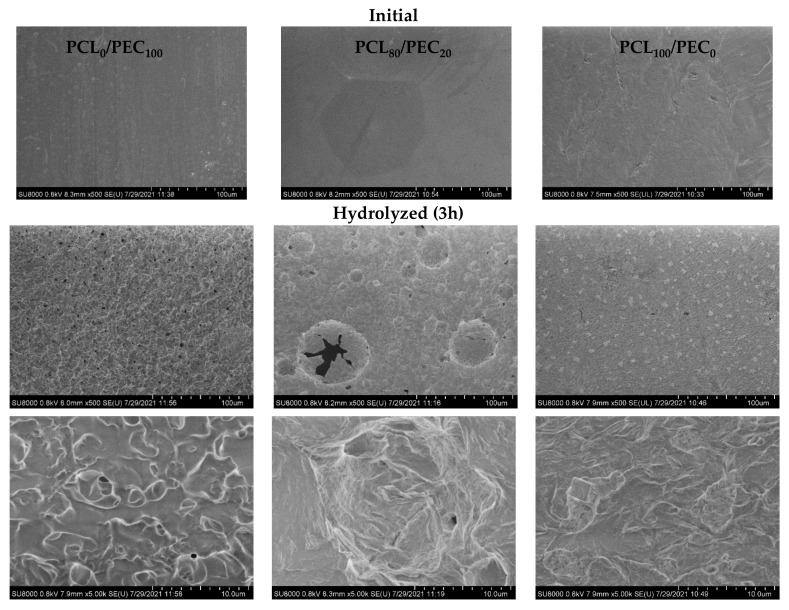
SEM micrographs of initial and hydrolyzed membranes after 3 h.

SEM micrographs of initial and hydrolyzed membranes after 3 h are shown in Figure 6. By comparison to initial samples, at the surface of hydrolyzed membranes, the presence of rounded holes and cracks must be noted in neat PEC, together with surface irregularities in a PCL_80_/PEC_20_ blend in contrast with neat PCL material that shows a slower degradation profile. These data are in accordance with the weight data loss obtained for alkaline degradation experiments, and clearly indicate the influence of PEC in order to control the rate of the hydrolysis process depending on the ratio of PCL and PEC polymers in the blends.

### 3.5. Contact Angle Measurements

One of the key factors in cell attachment and colonization is the hydrophilic character of polymer materials. In this way, highly hydrophobic blends would lead to inefficient cell colonization [30]. Therefore, achieving polymer blends with suitable cell attachment by varying their hydrophilic/hydrophobic characteristics is essential. On this basis, the water contact angle on the surface of pure PCL and PEC materials, together with their corresponding blends, was measured to evaluate the hydrophility variation of blends. The results obtained are compiled in Table 4.

It is well known that the contact angle measurements indicate the wettability of the material surface [31], so that a contact angle value above 90° corresponds to a hydrophobic surface. Pure PCL materials show values around 95°, which eventually could decrease the ability of cells to stick to the material’s surface [32]. Meanwhile, PEC shows a high hydrophilic character, with a contact angle of 40.5°. For the PCL/PEC blends, as result of increasing the percentage of PEC, the contact angle values decrease from 88.5° to 51.2° in PCL_90_/PEC_10_ and PCL_50_/PEC_50_, respectively, which clearly improves the surface wettability with respect to PCL.

### 3.6. Cell Response to PCL-PEC Films

#### 3.6.1. Cell Viability and Proliferation of MC3T3-E1 and hMSCs Cells Exposed to PCL/PEC Blends

Given the current interest in exploring the biomedical applications of new polymers-based biomaterials, the cell response of preosteoblasts and human mesenchymal cells was studied in the presence of PCL/PEC films by varying their composition.

**Figure 7:**
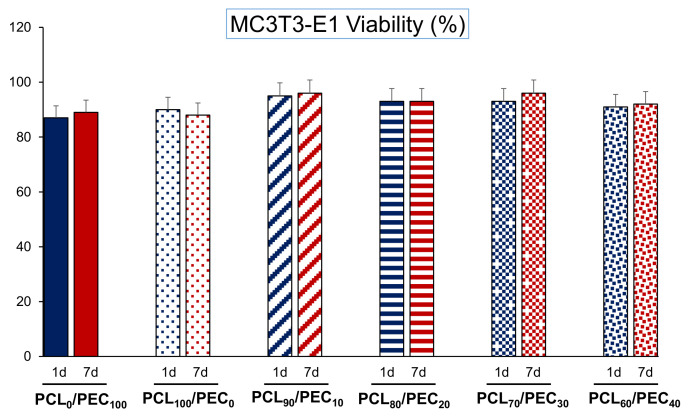
Cell viability of the MC3T3-E1 pre-osteoblasts cultured on PCL-PEC films during days 1 and 7.

**Figure 8:**
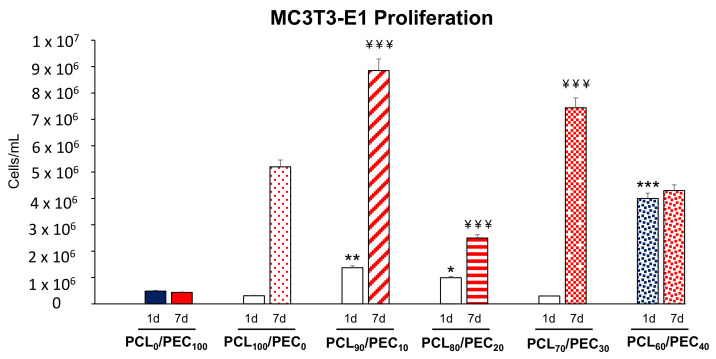
Cell proliferation (cells/mL) of the MC3T3-E1 pre-osteoblasts cultured on PCL/PEC films during 1 and 7 days. * *p* < 0.05, ** *p* < 0.01 and *** *p* < 0.005 vs. PCL_100_/PEC_0_ at 1 day of culture. ¥¥¥ *p* < 0.005 vs. PCL_100_/PEC_0_ at 7 days of culture.

**Figure 9:**
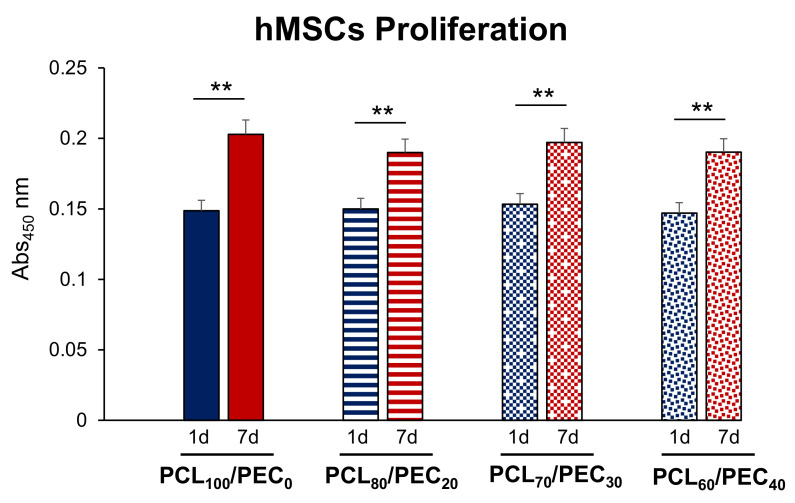
Cell proliferation (abs 450 nm) of the human mesenchymal cells (hMSCs) cultured on PCL/PEC films during days 1 and 7. ** *p* < 0.01, 1 vs. 7 days of culture.

Figure 7 displays the viability of MC3T3-E1 cells cultured on PCL/PEC films during days 1 and 7. On the other hand, Figure 8 and Figure 9 show the proliferation of preosteoblasts MC3T3-E1 and human mesenchymal cells, respectively, after 1 and 7 days of culture on the surface of the PCL-PEC films. With respect to MC3T3-E1 cellular viability, the results indicated very high viability percentages in all studied cases (PCL_90_/PEC_10_, PCL_80_/PEC_20_, PCL_70_/PEC_30_, PCL_60_/PEC_40_ and PCL_0_/PEC_100_ even), without any significant differences with respect to the control condition (~90% of viability), which correspond to cell cultures on the PCL_100_/PEC_0_ film.

Figure 8 shows that the MC3T3-E1 preosteoblast growth capacity after 1 day of culture on the PCL_90_/PEC_10_, PCL_80_/PEC_20_ and PCL_60_/PEC_40_ films was found to be significantly higher compared with that shown by these cells on the surface of the control film (PCL_100_/PEC_0_). After 7 days of culture, we also observed a significant increase in the preosteoblasts proliferation cultured on PCL_90_/PEC_10_, PCL_80_/PEC_20_ and PCL_70_/PEC_30_, compared with the proliferation obtained by MC3T3-E1 cells on the PCL_100_/PEC_0_ film. It is important to highlight that in all the cases studied, except for the PCL_0_PEC_100_ and PCL_60_/PEC_40_ films, the proliferation capacity of preosteoblasts significantly increased with culture time (from 1 to 7 days). The results obtained in the case of the PCL_0_/PEC_100_ film show the important delay caused by this polymer in the proliferation of this cell type. On the other hand, although the proliferation of preosteoblasts on PCL_60_/PEC_40_ films did not increase from day 1 to day 7 of culture, it is important to note that the levels of proliferation obtained on this film are similar to those obtained in the control condition (PCL_100_/PEC_0_).

The proliferation capacity of human mesenchymal cells is shown in Figure 9. No significant differences were observed in the proliferation of this cell type in all the tested PCL/PEC films compared with that obtained in the control condition (PCL_100_/PEC_0_), either at 1 or 7 days of culture. It is also important to mention that in all the cases studied, the proliferation capacity of human mesenchymal cells significantly increased with culture time (from 1 to 7 days).

#### 3.6.2. Cell Differentiation of MC3T3-E1 and hMSCs Cells Exposed to PCL/PEC Blends

**Figure 10:**
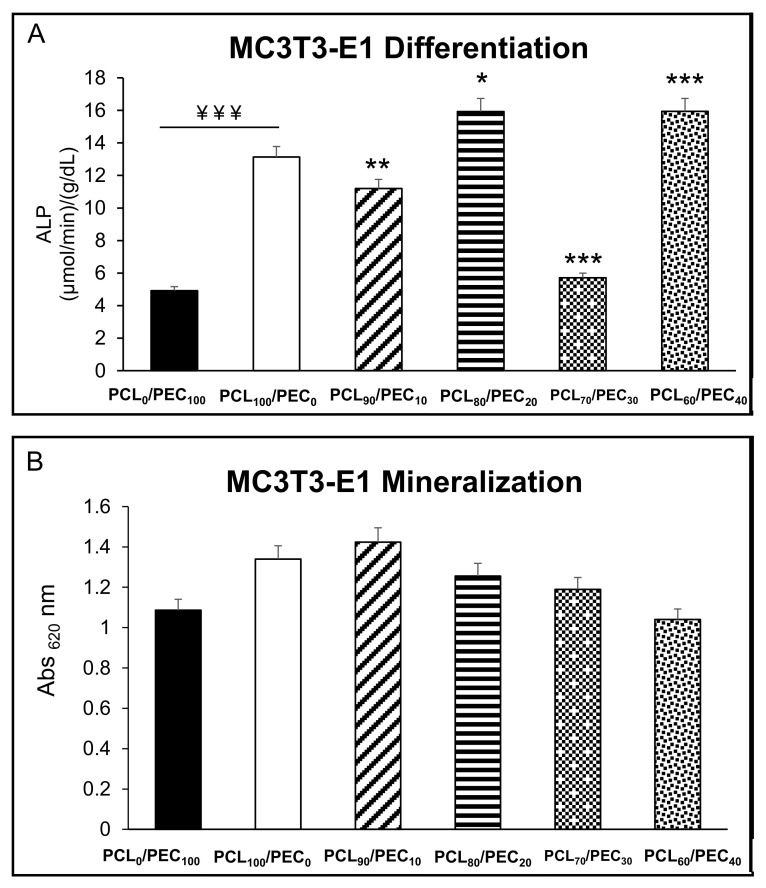
(**A**) Intracellular alkaline phosphatase (ALP) activity expressed as µmol/min/g/dL protein, normalized by total protein content, and (**B**) extracellular matrix mineralization of MC3T3-E1 preosteoblasts, after 7 and 10 days of culture, respectively, on PCL/PEC films. ¥¥¥ *p* < 0.005 PCL_0_/PEC_100_ vs. PCL_100_/PEC_0_. * *p* < 0.05, ** *p* < 0.01 and *** *p* < 0.005 vs. PCL_100_/PEC_0_.

Figure 10A shows the intracellular alkaline phosphatase activity of MC3T3-E1 preosteoblasts after 7 days of culture on the PCL/PEC films. Results reveal that there are important significant differences in the ALP activity of the MC3T3-E1 cells. Although the cells cultured on the PCL_80_/PEC_20_ and PCL_60_/PEC_40_ films show increased ALP activity, those cultured on the PCL_90_/PEC_10_ and PCL_70_/PEC_30_ films have decreased compared with that shown by cells cultured on the control film (PCL_100_/PEC_0_). Figure 10B displays extracellular matrix mineralization used as late marker of the osteoblast differentiation process. No significant differences were observed between all studied conditions relative to the formation of extracellular calcium phosphate deposits.

**Figure 11:**
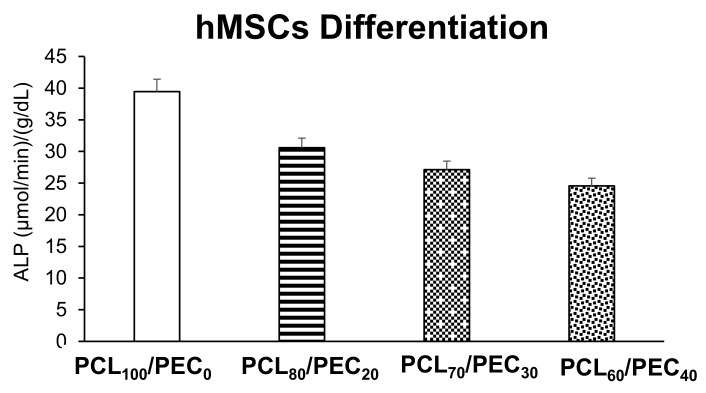
Intracellular alkaline phosphatase (ALP) activity expressed as µmol/min/g/dL protein, normalized by total protein content of human mesenchymal cells after 7 days of culture on PCL/PEC films.

Figure 11 displays the intracellular alkaline phosphatase activity of human mesenchymal cells after 7 days of culture on the PCL/PEC films. No significant differences were observed between all tested conditions compared with the control material (PCL_100_/PEC_0_). Despite this, a notable decrease in the intracellular ALP of hMSCs is observed as the percentage of PCL polymer in the films decreases.

## 4. Discussion

The FTIR analysis of neat polymers, PEC, and PCL, allowed us to identify their characteristic bands. It can be noted that if two polymers of the blend are compatible, this would be due to several chemical interactions, such us hydrogen bonding or dipolar interaction between the functional groups present in both polymers, which is reflected in band shifts or broadening of the peaks [33]. Then, the FTIR spectra of blends should show significant changes in their characteristic bands with respect to the spectra of the different components [34,35]. Here, it has been observed that the increasing addition of PCL in the blends was reflected in the progressive shift to lower wavenumber of PEC carbonyl peak, and when the PEC increased in the blend the PCL carbonyl peak shifted to a higher wavenumber. Additionally, the displacement of stretching bands of O-C-O and C-O-C groups in blends was observed with respect to the neat polymers. That effect would suggest intramolecular interactions between carbonyl groups of polymers and their compatibility in blends [5].

The thermal stability was studied by thermogravimetric analysis. The T_max_ was determined for neat PCL and PEC membranes at 398 °C and 210 °C, respectively, which agrees with previous reports, where the thermal degradation in the case of PEC is associated with the polymer principal chain unpacking [36]. For all blends, the T_max_ takes place between those of pure PCL and PEC. The addition of increasing the amount of PCL in blends improves the thermal stability of PEC, which is in accordance with results obtained by other authors concerning polycarbonate blends and the compatibility of their components [35]. Moreover, the interactions between PCL and PEC polymers that were described in FTIR analyses are therefore confirmed, something that is reflected in the compatibility of the PEC and PCL polymers.

The Tg of PEC was determined by differential scanning calorimetry analysis. It was seen to increase in PCL_50_/PEC_50_, and for higher percentages of PCL it was not negligible. It is associated with the lower amount of amorphous PEC in blends and the restricted chain mobility by the crystalline phase of PCL, which increased up to 47.5% in PCL_90_/PEC_10_. Additionally, the melting temperature (Tm) and crystallization temperature (Tc) of PCL decreased slightly with the addition of PEC. This effect would be related to the influence of the flexible PEC phase added in blends, which could affect both the crystallization temperature and the size of crystalline entities and support the miscibility of polymers, at least at the percentages used in this study. Those results are in accordance with AFM analysis, which showed the compatibility in the blends. The membranes exhibited low roughness and continuous morphology with the absence of microstructures and phase separation. The growth of PCL crystals was influenced by the incorporation of PEC in blends, and in the case of PCL_70_/PEC_30_, the crystal size decreases and smaller homogeneously dispersed crystalline entities are observed with respect to those in PCL_90_/PEC_10_.

Polymer surface wettability is known to affect cell attachment and it is one of the key factors in cell attachment and colonization. Cell adhesion to biomaterials is crucial for subsequent cellular processes to occur. Good cell adhesion is expected when the surface provides multiple cell-binding points, which enhances the surface of the cellular interfacial area [37]. To correlate the cell adhesion with the hydrophilicity of the membranes, their water contact angles were analyzed. A considerable decrease was observed from 94° to 40° for PCL and PEC, respectively, and their hydrophilicity was modified by blending the polymers. In this way, although PEC is a biocompatible and degradable polymer, highly hydrophobic blends would lead to inefficient cell colonization [30]. Therefore, achieving polymer blends with suitable cell attachment by varying their hydrophilic/hydrophobic characteristics is essential. In general, it can be observed that contact angle values range from 94° to 65° for neat PCL to PCL_60_/PEC_40_ blends. The addition of increasing amounts of PEC to blends obtained led to improvement in the hydrophilic character of blends and, finally, their biocompatibility. Otherwise, the hydrolytic degradation of PCL is usually low in the first stages [12,13], and it has been accelerated by adding PEC, which is almost completely hydrolyzed in alkaline solution after 96 h, which will improve its degradation and resorption kinetics in a physiologic environment. It is well known that a key parameter in tissue engineering is the degradation rate and the evolution of material properties during degradation, since the effect of changes in surface chemistry are crucial in cell adhesion and its integration with the host tissue with the purpose of finding a way to initiate the ideal condition of the degradation rate of related material similar to the regeneration rate of the tissue associated with the biomaterial in order to sustain the biomechanical integrity of the regenerating place, and to optimize the body repair response. Thus, two decisive crucial parameters are present in polymer blends after the addition of increasing PEC amounts: the enhancement of hydrophilicity together with the possibility of faster resorption by the organism of PCL/PEC blends.

Several biological parameters such as cell viability, proliferation, and differentiation in both MC3T3-E1 preosteoblasts and human mesenchymal cells were addressed to evaluate their cell response to different polymer membranes aimed at bone tissue regeneration. The MC3T3-E1 cell type is a murine calvaria-derived pre-osteoblastic cell line used as an archetypal model of in vitro osteoblast development [38]. Human mesenchymal stem cells are non-hematopoietic, multipotent stem cells with the capacity to differentiate into mesodermal lineage such as osteocytes, adipocytes, and chondrocytes, as well as ectodermal (neurocytes) and endodermal lineages (hepatocytes) when they are cultured in their specific differentiation mediums [39,40]. These two cell types have different characteristics in terms of expression of cell surface markers, long-term in vitro culturing, in vitro differentiation, among others, and for these reasons have been chosen for this comparative study.

Cell viability is an important biological parameter related to the integrity of the cell membrane, which allows evaluating the biocompatibility or cytotoxicity of any biomaterial. In addition to cell viability, another biological parameter widely used to test the cellular response to biomaterials is cell proliferation, which reflects the growth capacity of cells in the presence of such materials. With respect to the cellular proliferation, the results shown in this study highlight a different cell response to the polymer films depending on the cell type tested. Specifically, the MC3T3-E1 preosteoblast proliferation was affected depending on whether they are cultured on one polymeric film or another different blend composition. However, human mesenchymal stem cells proliferated in a similar way in all the films tested.

The osteoblastic cell line MC3T3-E1 is characterized by having high alkaline phosphatase (ALP) activity in the resting state. ALP is a glycoprotein present on the surface of the cell that is detected in the early stages of the differentiation process, and is involved in the mineralization process. These cells have also the capacity to differentiate into osteoblasts and osteocytes [41]. The differentiation process of osteoprogenitor cells is a critical stage for which premature osteoblasts (preosteoblasts) must be transformed into mature osteoblasts, starting with ALP expression and ending with mineralized nodule formation [42]. It has been already described by different authors that the cell differentiation is a complex process by which cells go through different cell stages; thus, a high cell proliferation rate is associated with low levels of intracellular ALP activity, and vice versa [43,44]. In this sense, our MC3T3-E1 preosteoblast results agree with the literature, as has been described above. Thus, the PCL_90_/PEC_10_ and PCL_70_/PEC_30_ films mainly favour the active replication of MC3T3-E1 cells (Figure 8), and hence, the intracellular ALP activity of these cells is low (Figure 9). On the other hand, higher ALP activity of preosteoblasts cultured on PCL_80_/PEC_20_ and PCL_60_/PEC_40_ films is related with their lower cell proliferation rate, Figure 10. hMSCs proliferated in a similar way on all the polymeric films tested. However, although no significant differences were observed, there is an evident tendency to decrease ALP as PCL content decreases. In this case, we could affirm that the PCL/PEC content of the different films studied mainly affects the ALP enzymatic activity. As shown, although MC3T3-E1 preosteoblasts present good cell viability when cultured on PCL_0_/PEC_100_ blends, in terms of proliferation, these values show the worst data, which agree with the lower values of the contact angle. Regarding PCL/PEC blends, general improvements in proliferation and differentiation data can be observed, that imply that PEC addition to blends enhances cell biocompatibility. This finding indicates that PCL/PEC blends appear to affect the osteoblastic maturation by up-regulating local cellular processes in response to cell–material interactions. With respect to human mesenchymal stem cells, the proliferation and differentiation results obtained with the different PCL/PEC blend shows similar results in comparation with neat PCL_100_/PEC_0_ blends, although it should be noted that the results obtained show that PEC addition improves the hydrolytic degradation of materials, as already seen.

Overall, it is important to highlight the obtention of materials with good compatibility and homing properties to promote bone cell growth. In this regard, the interactions of biomaterials with bone tissue involve a broad range of cellular events, which are closely related to several parameters that will be described. Thus, it is well known that osteoblasts, which must be differentiated from their precursors, are cells that play important roles in bone tissue repair since they increase bone growth at the defect area by synthesizing the bone matrix, which is subsequently mineralized [45], as shown in the works of Khotib et al. [46,47], where biomaterials based in hydroxyapatite (HA) that promote bone cell growth are described. These are osteoinductive, and can induce osteogenesis, and thus, new bone growth. However, there are other parameters that influence osteoinductive properties. In terms of mechanical properties, it is key to obtain materials with a similar compressive and tensile strength to that of human bones, which translates into the achievement of manageable materials as described above in the characterization of materials. Against this background, there are several factors which can be modulated, such as surface chemistry, surface topography (including roughness and patterning), wettability and surface mechanical characteristics. These parameters will have a decisive influence in the cell–matrix interactions and subsequent responses [48]. In this work, two dimensional materials haven been constructed, i.e., as polymer surfaces colonized by cells, through non-specific cell–material interactions, through the so-called weak chemical bonding, such as, for example, hydrogen bonding or another interaction (electrostatic, polar, or ionic) between molecules on cell membrane and functional chemical groups on the polymers, and with an adjustment of their surface wettability, as shown in the results obtained by contact angle measurements [49].

Additionally, in relation to homing properties, the surface roughness is a parameter which can be adjusted. For example, in relatively rough surfaces, cell adhesion is relatively low and irregular due to the presence of irregularities on the surface. In contrast, in more polished surfaces, the number of initially adhered cells, as well as its further growth, significantly increased [50,51]. However, otherwise, very smooth surfaces cannot guarantee a firm and strong cell adhesion. This is in agreement with the results obtained by AFM in roughness values for the polymer blends obtained. Taking into consideration the surface polarity and wettability, the parameters of the surface roughness also have their optimum range, according to the type of material and the type of cells. In this manner, although the height and depth of the surface irregularities and distances between them will be decisive, their shape, especially their sharpness, must be taken into consideration as it may mechanically damage the cells.

Therefore, the results presented in this study show that distinct cell types respond differently to the same biomaterial; thus, special care must be taken when choosing one cell type or another when it comes to studying the biocompatibility of any new material.

Considering all the data, we can say that these new polymer blends based in PCL/PEC are biocompatible, non-cytotoxic, and promote cell proliferation and differentiation. Due to the adequate cellular response obtained, this makes them promising candidates as biomaterials. Once their properties have been evaluated by in vitro assays, there are enough results to raise their potential application in tissue engineering and bone regeneration in a second stage of “in vivo” analysis. In this sense, the future research lines will be focused on study of their real applicability as biomaterials for tissue engineering in bone regeneration. For this purpose, in a later work, scaffolds will be made by 3D forming with the mixtures that have shown the best results, i.e., with PEC percentages between 30% and 10%. In this sense, when a new material is proposed as a potential candidate for a certain biomedical application, the in vitro methodology allows scientists to evaluate various biological phenomena in specific cells without potentially confounding variables present in whole organisms. In vitro testing is a straightforward research methodology, with a relatively low cost and faster time than the in vivo tests. Moreover, researchers can perform more detailed analyses and examine biological effects in a larger number of in vitro replica than they would in animal or human trials, which would be related to the regulations in experimental models.

## 5. Conclusions

New PCL/PEC blends have been obtained for the first time as “in vitro” proof of the concept of the biocompatibility of this type of new blend. A high miscibility and thus, compatibility, of these polymers has been observed, obtaining homogeneous blends in a simple way by casting. All blends obtained have been characterized by infrared spectroscopy (FTIR), confirming their good compatibility. Their thermal properties have been evaluated, and the variation in the material properties with the addition of PEC in terms of hydrophilicity has been analyzed by means of contact angle and surface roughness measurements (AFM). In addition, a study of the alkaline hydrolysis of these compared with PCL alone has been carried out. It has been observed that the addition of different percentages of PEC in the PCL mixtures allows us to obtain homogeneous mixtures and allows us to modify decisive parameters when evaluating their biocompatibility, such as increasing the hydrophilicity of the materials while maintaining a moderate roughness, so that materials with tailor-made properties can be obtained depending on the application. In this way, new blend with tunable properties is obtained in the easy way, with the aim of improving their biological properties in terms of an adequate cellular response.

In relation to the results obtained in the cell viability of the blends, it is observed that the presence of PEC in the membranes favors cell proliferation, which reflects the growth capacity of cells. With respect to the cellular proliferation, the results shown in this study show a different cell response to the polymer films depending on the cell type tested. On one hand, the MC3T3-E1 preosteoblast proliferation was affected depending on blend composition, but with better results compared with PCL neat polymers. However, hMSC cells proliferated all the films tested in a similar manner, but with an improvement in the hydrolytic degradation of blends and hydrophilic character.

With all these results in mind, we can conclude that these new materials could be useful for tissue engineering, although more experiments are needed following this preliminary “in vitro” study.

## Figures and Tables

**Table 1:** Summary of membrane compositions used in this work, along with optimized solution conditions in order to achieve a homogeneous mixture that will subsequently lead all membranes by casting method.

Membrane Composition (Casting at rt.)	wt% PCL (Added to PEC Solutions)	wt% PEC (Solution in 1.5 mL of Chloroform)	Weight of PEC (mg)	Weight of PCL (mg)
**PCL_0_/PEC_100_**	0	100	200	0
**PCL_50_/PEC_50_**	50	50	100	100
**PCL_60_/PEC_40_**	60	40	80	120
**PCL_70_/PEC_30_**	70	30	60	140
**PCL_80_/PEC_20_**	80	20	40	160
**PCL_90_/PEC_10_**	90	10	20	180
**PCL_100_/PEC_0_**	100	0	0	200

**Table 2:** Thermogravimetric data for all membranes, the temperature at 5 wt% weight loss (T_5_) and the maximum decomposition temperature (T_max_) obtained from the derivative weight loss curve (DTGA).

Membrane	T_max_ (°C)	T_5_ (°C)
**PCL_0_/PEC_100_**	210/--	203
**PCL_50_/PEC_50_**	225/393	209
**PCL_60_/PEC_40_**	226/394	216
**PCL_70_/PEC_30_**	227/395	220
**PCL_80_/PEC_20_**	244/396	237
**PCL_90_/PEC_10_**	258/397	253
**PCL_100_/PEC_0_**	--/398	359

**Table 3:** DSC data obtained in terms of melting temperature (Tm), crystallization temperature (Tc) and glass transition temperature (Tg). % Crystallinity index was obtained using the standard enthalpy of 100% crystalline of PCL (ΔH_m_^0^ = 139.5 J/g) [29].

Membrane	T_g_ (°C)	T_m_ (°C)	T_c_ (°C)	Crystallinity Index (%)
**PCL_0_/PEC_100_**	11	--	29.2	--
**PCL_50_/PEC_50_**	16	61.2	28.8	19.5
**PCL_60_/PEC_40_**	--	61.9	28.5	23.4
**PCL_70_/PEC_30_**	--	61.7	27.3	26.2
**PCL_80_/PEC_20_**	--	62.2	26.7	31.9
**PCL_90_/PEC_10_**	--	63.1	26.5	47.5
**PCL_100_/PEC_0_**	--	63.7	--	50.7

**Table 4:** Contact angle values measured for all materials obtained. Values were obtained using milliQ water as a solvent, from the media of six measures.

Membrane	Contact Angle Value
**PCL_0_/PEC_100_**	40.5 ± 0.9
**PCL_50_/PEC_50_**	51.2 ± 0.7
**PCL_60_/PEC_40_**	65.5 ± 0.8
**PCL_70_/PEC_30_**	75.7 ± 1.1
**PCL_80_/PEC_20_**	81.3 ± 0.9
**PCL_90_/PEC_10_**	88.5 ± 0.4
**PCL_100_/PEC_0_**	94.3 ± 0.6

## Data Availability

Not applicable.
